# Editorial: Radiation therapy using MRI-LINAC - the right way to start: a guide for physicians and physicists, volume II

**DOI:** 10.3389/fonc.2024.1487081

**Published:** 2024-10-17

**Authors:** Raphael M. Pfeffer, Enis Oyzar, Merav A. Ben David

**Affiliations:** ^1^ Assuta Medical Center, Tel-Aviv, Israel; ^2^ Acıbadem University, Istanbul, Türkiye

**Keywords:** stereotactic ablation body radiation therapy, MRgRT, radiation, oligometa, central lung tumors

The second volume in this important series on applicating the novel MR guided radiotherapy technique follows on from the previous volume.

Editorial: Radiation therapy using MR-linac: Further studies

Image-guided radiation therapy (IGRT) based on 3-dimensional imaging has had a major impact on the advancement of radiotherapy in the past 50 years. Soon after the clinical introduction of computerized tomography (CT) it was employed in radiotherapy, in order to visualize internal organs and to calculate the volumes of the tumor target and internal organs close to the tumor which could be affected by the radiation dose (1). 3D imaging enabled the first studies of extracranial extreme hypofractionation, commonly called stereotactic body radiation therapy (SBRT) or stereotactic ablative radiation therapy (SABR). Timmerman et al. pioneered SBRT in early stage lung cancer (2). The success of SABR for lung cancer was made possible by the ability to accurately identify the tumor on CT imaging of the thorax. Further incremental improvements came with real-time imaging on the radiotherapy (RT) treatment table (e.g cone beam CT) ensuring control and accurate delivery of SBRT plans.

The application of SABR to mediastinal, abdominal and pelvic sites requires accurate and precise imaging of these areas as provided by magnetic resonance imaging (MRI). The first commercially available clinical MRI radiotherapy system began treating patients on a radioactive cobalt-based system in 2014 (3). Over the last 10 years there have been gradual developments in the delivery of MR guided RT, as the successful incorporation of Linac-based radiation into the ViewRay system and the development of rival machines such as the Elekta Unity. There are now over 100 installations of MR guided RT systems in the world. A PubMed search shows a steady increase in publications regarding MR Linac reaching over 200 per year in recent years.

This volume is the second in a series of frontiers dedicated to advances in the clinical use of MR guided radiotherapy. The articles can be divided into 2 overlapping broad categories, clinical experience and physics with quality assurance.

A single institution series of pancreatic cancer patients receiving MRgSBRT to the pancreas with 5- 10 Gy fractions shows the range of patients being treated today. The great majority have locally advanced inoperable disease. The series includes patients with locally recurrent disease following prior surgery or fractionated radiation and a few patients with oligo-progressive disease treated to both the primary and the oligometastasis. One of the difficulties of treating pancreatic tumors with MRgSBRT is the visualization of surrounding small bowel and real-time changes in organs at risk around the target tumor. Another paper reports on improved inter-observer contouring accuracy of pancreatic cancer MRI with the use of butylscopolamine prior to MRI.

A major advantage of MRguided radiotherapy is the ability to have real time tumor tracking to ensure coverage of the target and avoid toxicity of adjacent organs. Two papers describe novel methods of tracking. A case report of a nodal recurrence of endometrial cancer used real time tracking of isodose lines to deliver daily isotoxic doses. A single institute series of intrathoracic tumors who received MRgSBRT due to proximity to organs at risk showed the importance of real time tracking. A further study looked at gating protocols for apical lung lesions. Cardiac lesions close to the right ventricles are especially difficult to treat with focal radiotherapy and in this series there is a report of 2 patients safely treated with a dose of 30 Gy in 3 fractions of 10 Gy each.

A couple of papers described aspects in dosimetry of MR guided SBRT. One paper looked at techniques of delineation of gas packets and the impact of these gas packets using various dose and gas volume measurement protocols. Another paper reported on the benefits of online adaptation in the treatment of prostate cancer with MRgSBRT.

The addition of non-proprietary software upgrades to MRgRT planning programs requires meticulous QA. An online QA program to evaluate such upgrades is described.

In summary, the several papers in this volume describe a wide range of issues involved in implementing MRgSBRT and serve as an important addition to existing knowledge.
